# Genomic and evolutionary relationships among wild and cultivated blueberry species

**DOI:** 10.1186/s12870-023-04124-y

**Published:** 2023-03-06

**Authors:** Byron R. Manzanero, Krishnanand P. Kulkarni, Nicholi Vorsa, Umesh K. Reddy, Purushothaman Natarajan, Sathya Elavarthi, Massimo Iorizzo, Kalpalatha Melmaiee

**Affiliations:** 1 Department of Agriculture and Natural Resources, Delaware State University, Dover, DE 19901 USA; 2 Department of Plant Biology, Rutgers University, New Brunswick, NJ 08901 USA; 3 Philip E. Marucci Center for Blueberry and Cranberry Research and Extension, Chatsworth, NJ 08019 USA; 4 Department of Biology, West Virginia State University, Institute, WV 25112 USA; 5 Department of Horticultural Science and Plants for Human Health Institute, NC State University, Kannapolis, NC 28081 USA

**Keywords:** Blueberry, Genotyping-by-sequencing, Single nucleotide polymorphisms, Principal component analysis, Admixture analysis, Domestication

## Abstract

**Background:**

Blueberries (*Vaccinium* section *Cyanococcus*) are an economically important fruit crop in the United States. Understanding genetic structure and relationships in blueberries is essential to advance the genetic improvement of horticulturally important traits. In the present study, we investigated the genomic and evolutionary relationships in 195 blueberry accessions from five species (comprising 33 *V. corymbosum*, 14 *V. boreale,* 81 *V. darrowii,* 29 *V. myrsinites,* and 38 *V. tenellum*) using single nucleotide polymorphisms (SNPs) mined from genotyping-by-sequencing (GBS) data.

**Results:**

GBS generated ~ 751 million raw reads, of which 79.7% were mapped to the reference genome *V. corymbosum* cv. Draper v1.0. After filtering (read depth > 3, minor allele frequency > 0.05, and call rate > 0.9), 60,518 SNPs were identified and used in further analyses. The 195 blueberry accessions formed three major clusters on the principal component (PC) analysis plot, in which the first two PCs accounted for 29.2% of the total genetic variance. Nucleotide diversity (π) was highest for *V. tenellum* and *V. boreale* (0.023 each), and lowest for *V. darrowii* (0.012). Using TreeMix analysis, we identified four migration events and deciphered gene flow among the selected species. In addition, we detected a strong *V. boreale* lineage in cultivated blueberry species. Pairwise SweeD analysis identified a wide sweep (encompassing 32 genes) as a strong signature of domestication on the scaffold VaccDscaff 12. From this region, five genes encoded topoisomerases, six genes encoded CAP-gly domain linker (which regulates the dynamics of the microtubule cytoskeleton), and three genes coded for GSL8 (involved in the synthesis of the cell wall component callose). One of the genes, *augustus_masked-VaccDscaff12-processed-gene-172.10*, is a homolog of *Arabidopsis* *AT2G25010* and encodes the protein MAINTENANCE OF MERISTEMS-like involved in root and shoot growth. Additional genomic stratification by admixture analysis identified genetic lineages and species boundaries in blueberry accessions. The results from this study indicate that *V. boreale* is a genetically distant outgroup, while *V. darrowii*, *V. myrsinites,* and *V. tenellum* are closely related.

**Conclusion:**

Our study provides new insights into the evolution and genetic architecture of cultivated blueberries.

**Supplementary Information:**

The online version contains supplementary material available at 10.1186/s12870-023-04124-y.

## Background

Blueberries are classified under the genus *Vaccinium* sect. *Cyanococcus,* a member of the Ericaceae family, and encompasses approximately 400-500 shrubs/small tree species globally [1, 2]. They exist at different ploidy levels, including diploids (2*n* = 2x = 24), tetraploids (2*n* = 4x = 48), and hexaploids (2*n* = 6x = 72). *Vaccinium* genus includes multiple relevant berry crops, the most important being blueberry (*V. corymbosum* L.) and cranberry (*V. macrocarpon* Ait.). However, the evolution and systematics of *Vaccinium* are poorly understood, probably because of extensive hybridization and wide morphological variability.

*V. corymbosum* is the prime source of germplasm in cultivated highbush blueberry [3]. Domesticated *V. corymbosum* collections have been used extensively in breeding programs. During the last 50 years, several southern species were hybridized with *V. corymbosum* to develop the southern highbush (SHB) cultivars, specifically to lower the number of chilling hours required to flower and adapt to the warmer climates [4–6]. Such breeding efforts produced a range of interspecific hybrids, thus reducing the genetic contribution of *V. corymbosum* from an average of 97% in the original northern highbush (NHB) blueberry accessions to 72% in the average SHB cultivar [7]. Commercial blueberry varieties have also been derivatives of wide hybridization for developing homoploid hybrids with desirable traits. Recently developed hybrids are the result of a cross between domestic cultivars and wild species to attribute a wider genetic variance to these plants with hopes for plants to inherit superior traits [8]. Heteroploid crosses are partially fertile, which offers the possibility of introgression into cultivated germplasm. Blueberries are recently domesticated perennial fruit-bearing plants, and their popularity has been growing because of their palatability and the health benefits associated with their consumption [9]. Blueberries possess several essential vitamins, secondary metabolites, antioxidants (e.g., anthocyanins, flavonols, hydroxycinnamic and hydroxybenzoic acids, and proanthocyanins [10].

Blueberry cultivars can be grouped according to their low-temperature requirement as high, moderate, and low chilling. Subjecting perennial plants to chilling conditions for an extended period stimulates post-winter flowering initiation [9]. When first domesticated, blueberries were predominantly grown in the northern parts of the United States, possibly because of the suitable edaphoclimatic conditions that promoted the growth of plants, such as pH 4.8 and temperatures of 0–7 °C [11]. Increasing temperatures, droughts, and adverse weather conditions significantly affect blueberry production [12]. Drought is a major factor in decreasing yields, and high temperatures can negatively affect pollination and fruit development. High atmospheric UV levels also can negatively affect blueberry production and fruit quality [4, 13, 14].

Interspecific hybridizations with wild southern lowbush species led to varieties that are tolerant to diverse climates [4], thus allowing them to maintain their average yield and fruit quality (SHB blueberries). Consistent increase in atmospheric temperatures over the past few decades might have enhanced adaptation to higher temperature stress in the fruit development phase, an absolute necessity to keep or increase the world’s blueberry production. These traits can be introgressed from diverse species, such as *V. darrowii* or *V. tenellum* native to neotropical regions or *V. myrsinites,* which is a tetraploid [1].

Blueberry was not cultivated until the early 1900s, so it is one of the most recent berry crops to be domesticated. In 1908, Dr. F.V. Coville, a US Department of Agriculture researcher, studied wild blueberries and selected plants bearing large-sized berries for breeding. Later, he made crosses among the best to develop the first 15 commercial varieties of blueberries. The “Bluecrop,” “Blueray” and “Earliblue” varieties that he developed are still widely grown and highly popular cultivars today. Thus, he revolutionized blueberry production. Several NHB and SHB cultivars are currently cultivated across the United States, Canada, and many other countries. Several researchers have established genetic relationships among the wild and cultivated blueberry species using morphological characteristics and intercrossability.

Since the identification of advanced molecular markers, the population genetic structure of diploid and tetraploid blueberries has been addressed using various DNA marker systems [15–21]. Understanding blueberry evolution, migration events, and species boundaries between wild blueberry species are essential to continue breeding new cultivars for environmental adaptability while maintaining genetic diversity [22]. These boundaries are governed by gene flow and speciation. Shared polymorphisms define species boundaries that could be sympatric and allopatric [23]. *Vaccinium* species from the *Cyanococcus* section are widespread throughout eastern North America, striving in diverse environments, thus widening the genetic divergence among the species [5, 24]. Wild species in the neotropics and subtropics generally tolerate heat and drought [8]. These wild species could be a repository of favorable alleles for future use in introgression programs. Recently, we have shown that the southern species *V. darrowii* exhibits a differential response of morpho-physiological and molecular mechanisms compared to the *V. corymbosum* plants under heat stress [25]. Thus, analyzing the genetic structure of species can help identify diverse lineages and play an important role in breeding.

Reduced representation libraries combined with next-generation sequencing have combined SNPs and scoring methods in a single process to identify diversity-targeted studies [26] effectively. Genotyping-by-sequencing (GBS) resolves genomic level differences that can be converted into markers that can be used for several downstream applications [26, 27]. Large genomic datasets can now be used to analyze the genetic structure of a population, species evolution, genetic lineages, and selection signals. In the present study, we selected 33 highbush (northern and southern) *V. corymbosum* accessions and 162 clones representing four blueberry species — *V. boreale* from a northern region versus *V. darrowii, V. tenellum,* and *V. myrsinites* from a southern region — to understand their genetic relationships and level of inter-species admixture. The selected blueberry accessions/species were sequenced using GBS, and high-quality SNPs were used to detect the genetic lineages and identify species boundaries among the selected blueberry accessions. In addition, we revealed migration events and identified strong selection signals related to domestication in blueberry.

## Results

### GBS summary

GBS of 195 blueberry accessions yielded ~ 751 million raw reads of 75 bp long (Table 1). The average number of raw reads per sample was 3.7 million. Barcode tags with a minimum of three read counts were used for SNP calling. The *V. corymbosum* cv. Draper v1.0 reference genome was used for read mapping [28]. Specifically, we used the 12 scaffolds representing each blueberry chromosome’s most extended haplotype. About 588 million reads (an average of 2.9 million reads per sample) were mapped to the reference genome sequence. The overall mapping percentage to the reference genome was 79.7% (Table 1).

**Table 1:** GBS summary showing reads and mapping percentage

	Number of Sequencing Reads with Barcode	Total number reads mapped to the Tetraploid Blueberry genome	Mapping %
Total	750,806,666	588,679,572	
Average per sample	3,716,864	2,914,255	79.72

The SNPs were filtered by using 1) read depth, DP > 3, 2) MAF > 0.05, and call rate > 0.9. After quality filtering and trimming, 60,518 SNPs were obtained from the sequencing data. The average number of SNPs per kilobyte of genome length ranged from seven to 10. The scaffold VaccDscaff 1 had the highest number of SNPs (6200), and the scaffold VaccDscaff 12 had the lowest (4091) (Table 2).

**Table 2:** Scaffold-wise summary of single nucleotide polymorphism (SNP) statistics from genotyping-by-sequencing analysis of 195 blueberry accessions

Chromosome	Chromosome Length (bp)	Filtered SNPs (MAF > 0.05; call rate 90; DP > 3)	Average number of filtered SNPs per kb genome length
Total number of taxa		195	
Total number of SNPs		60,518	
VaccDscaff 1	46,295,995	6200	7
VaccDscaff 2	44,818,276	5624	8
VaccDscaff 4	42,981,373	4871	9
VaccDscaff 6	42,795,824	4457	10
VaccDscaff 7	41,705,179	4918	8
VaccDscaff 11	40,122,599	5614	7
VaccDscaff 12	39,741,682	4091	10
VaccDscaff 13	39,652,356	5104	8
VaccDscaff 17	38,874,919	5004	8
VaccDscaff 20	37,996,905	4409	9
VaccDscaff 21	37,975,728	5001	8
VaccDscaff 22	37,315,645	5225	7

*M.A.F.* minor allele frequency, *D.P.* read depth

### Principal component analysis

**Fig. 1:**
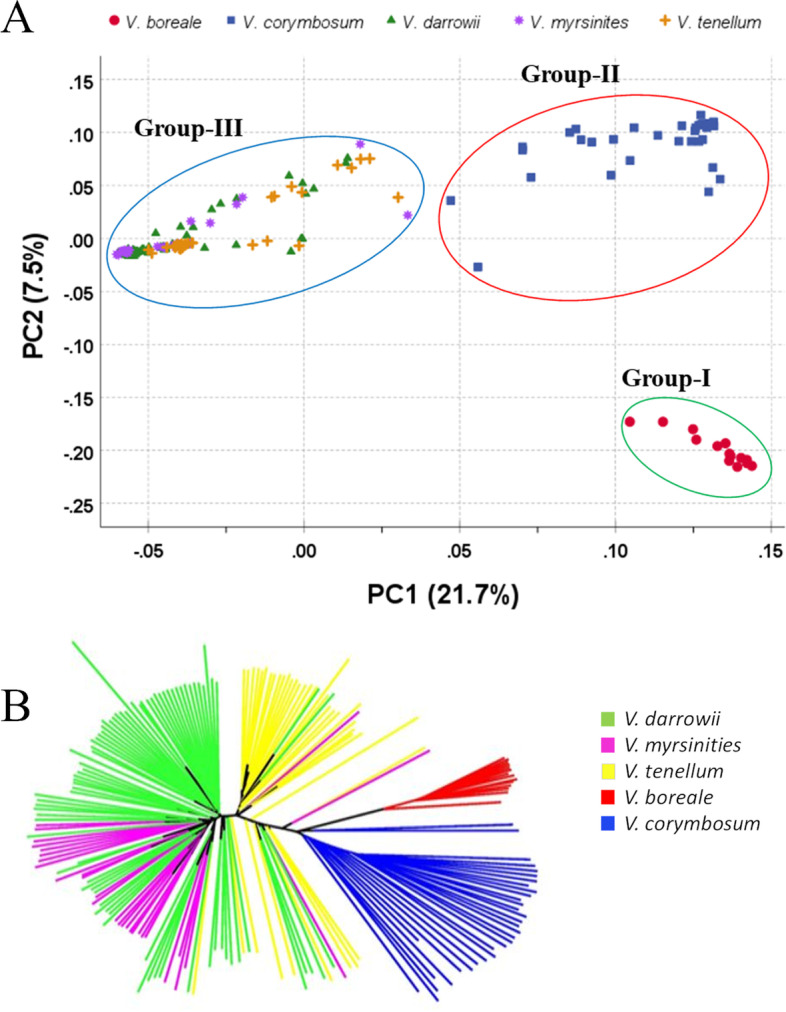
**A** The first and second components of the principal component analysis of 195 blueberry accessions. **B** Phylogenetic tree of 195 blueberry population (163 wild and 32 cultivated species)

A total of 60,518 SNPs were used in PCA for genetic differentiation of the selected blueberry accessions. The first two principal components (PCs) accounted for 29.2% of the total genetic variation (axis X = 21% and axis Y = 6.9%). All 195 blueberry accessions formed three major clusters on the PCA plot (Fig. 1A). Group I included all 14 *V. boreale* accessions, whereas group II included all 33 *V. corymbosum* accessions*,* which represent the cultivated pool used in the study*.* The remaining 148 accessions belonging to *V. darrowii*, *V. myrsinites*, and *V. tenellum* clustered into group III. Group I, comprising *V. boreale* species, was the most distant species. In contrast, groups II and III were closer to each other than to group I. Within the *V. corymbosum* group, the accessions were tetraploid except for NJOPB-8, which was diploid and was separated from the tetraploid *V. corymbosum* cluster (Fig. 1A).

To understand how *V. boreale, V. darrowii*, *V. myrsinites*, and *V. tenellum* species are genetically related to *V. corymbosum,* we performed a set of focused PCA, which included only polymorphic SNPs from the subsets of Taxon. The first two PCs for *V. corymbosum* vs *V. boreale, V. corymbosum* vs *V. darrowii, V. corymbosum* vs *V. myrsinites,* and *V. corymbosum* vs *V. tenellum* accounted for 6.5, 15.5, 8.9, and 9.2% of the genetic variation, respectively (Fig. S1). PCA separated individual species and formed two major clusters, one with cultivated *V. corymbosum* and the other with sub-clusters of wild species. Because the objective of this study was to identify bridge accessions for use in introgression, we co-located NJ87-29-23, a known *V. corymbosum* accession, to be shared between the clusters of *V. boreale* vs *V. corymbosum* (Fig. S1). In addition, three *V. corymbosum* cultivars, ‘Biloxi’, ‘Sunshine blue’, and ‘Pink Lemonade’, were distant from the remaining *V. corymbosum* cultivars.

### Genetic diversity and population differentiation

We estimated nucleotide diversity to assess genetic variation among the accessions of each species (Table 3). Genome-wide nucleotide diversity was highest for *V. tenellum* and *V. boreale* (0.023 each) and lowest for *V. darrowii* (0.012). To understand the degree of genetic differentiation between the selected blueberry accessions, we used *F*_*ST*_ analysis with 95% confidence intervals. The highest pairwise genetic differentiation was between *V. myrsinites* and *V. boreale* (*F*_*ST*_ = 0.42), and the lowest was between *V. myrsinites* and *V. darrowii* (*F*_*ST*_ = 0.022) (Table 4), which indicates *V. darrowii* as a sister species of *V. myrsinites*.

**Table 3:** Intraspecific nucleotide diversity in blueberry populations

	*p*-value	Nucleotide diversity
*V. tenellum*	0.000171	0.023
*V. myrsinite*s	0.000107	0.015
*V. darrowii*	0.000498	0.012
*V. corymbosum*	0.000134	0.015
*V. boreale*	2.853E-05	0.023

**Table 4:** Genome-wide pairwise fixation index (*F*_*ST*_) analysis for the blueberry species in this study

Species	***V. tenellum***	***V. myrsinites***	***V. darrowii***	***V. corymbosum***	***V. boreale***
***V. tenellum***	0.000	0.086	0.061	0.227	0.392
***V. myrsinites***	0.086	0.000	0.022	0.252	0.420
***V. darrowii***	0.061	0.022	0.000	0.264	0.404
***V. corymbosum***	0.227	0.252	0.264	0.000	0.244
***V. boreale***	0.392	0.420	0.404	0.244	0.000

The genome-wide window-based *F*_*ST*_ analyses between the species (Fig. S2) showed signatures of positive selection in pairwise comparisons. These SNPs might be the regions of positive selection and may be necessary for domestication or genetic improvement. However, pairwise *F*_*ST*_ analyses of *V. boreale* with all the other species indicated negative selection, so it may be an ancestral species and underwent genome-wide changes. We identified 17 SNPs spanning eight scaffolds with the highest *F*_*ST*_ (Table S1). These SNPs could be helpful to locate negative selection footprints.

### Phylogenetic analysis

The genetic relationships of the selected 195 blueberry accessions are illustrated in the neighbor-joining (NJ) tree in Fig. 1B. The 195 accessions clustered into three separate groups: 1) *V. corymbosum*, 2) *V. boreale*, and 3) a group with a mixture of *V. darrowii, V. myrsinites*, and *V. tenellum* accessions. The southern wild species formed a single cluster regardless of their ploidy. The phylogenetic clustering analysis indicated that *V. darrowii, V. myrsinites,* and *V. tenellum* do not have clear species boundaries revealing reticulate evolution. The results of the NJ tree were highly consistent with those of PCA (Fig. 1A).

To identify sub-populations within species and estimate genetic relationships within them, NJ trees were developed separately for each species (Fig. S3). *V. darrowii, V. myrsinites,* and *V. tenellum* accessions were divided into sub-populations, each separated into three sub-clusters, whereas *V. boreale and V. corymbosum* accessions were each grouped into two clusters. In the *V. corymbosum,* NHB formed a separate group, but 6 NHB accessions grouped along with the SHB group. Thus, there was no clear separation of SHB accessions from all the NHB accessions.

### Admixture analysis

**Fig. 2:**
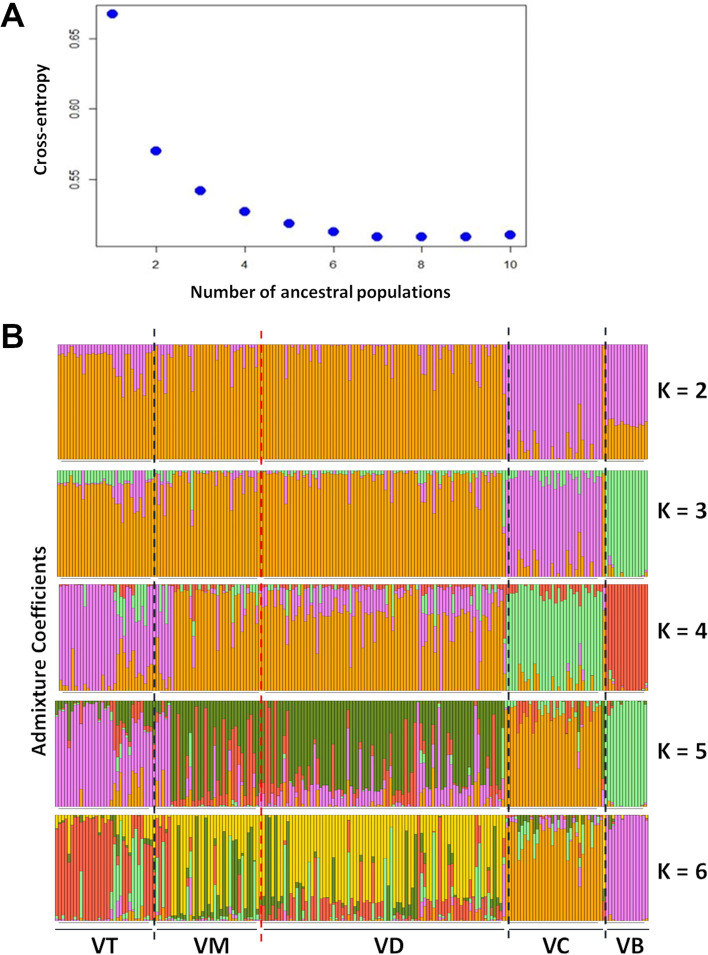
Lineage sorting of the blueberry species based on the admixture analysis. **A** Cross-entropy curve depicting the delta K values ranging from K2 to K10. The flat curve was observed after K = 6. **B** Admixture proportion revealed for each blueberry species inferred with the R package LEA. Each vertical bar represents an individual blueberry species. VT: *V. tenellum*; VM: *V. myrsinites*; VD: *V. darrowii*; VC: *V. corymbosum*; VB: *V. boreale*

Principal component analysis (PCA) and NJ trees singled out *V. corymbosum* and *V. boreale* accessions as separate groups and differentiated them from *V. tenellum*, *V. darrowii,* and *V. myrsinites* accessions. For genetic differentiation among *V. tenellum*, *V. darrowii,* and *V. myrsinites* accessions, we used admixture analysis with the Landscape and Ecological Association Model [29]. We applied a 10-factor replication to detect clustering (K = 1:10) and performed the analysis when the parameter K was between 2 and 6 (Fig. 2A). At K = 2, the accessions comprising *V. corymbosum* and *V. boreale* were first separated (with one exception) from the southern wild accessions and formed subgroup I (Fig. 2B). At K = 3, *V. boreale* accessions were separated from subgroup I and formed subgroup III. Therefore, the *V. boreale* genome seems to be distinct from the other species included here. At K = 4, subgroup II was further divided into subgroup V (comprising *V. tenellum* accessions) and subgroup VI (comprising *V. darrowii* and *V. myrsinites* accessions), with some degree of admixture. At K = 5 and K = 6, the *V. darrowii,* and *V. myrsinites* accessions did not form subgroups despite differences in the ploidy levels, which indicates that these two species are highly admixed. Cross-entropy curve (Fig. 2A) suggested that K-4 showed a plateau of the cross-entropy curve, indicating a statistically significant lineage pattern. Thus, the population can be grouped into four subpopulations. These results are primarily by the phylogenetic and PCA analysis. Further, the study suggested that very few accessions may have a highly homogenous genetic background, and some admixture exists in several accessions, which may be due to the high frequency of interspecific hybridization that is common in the *Vaccinium* genus [21].

### TreeMix analysis

**Fig. 3:**
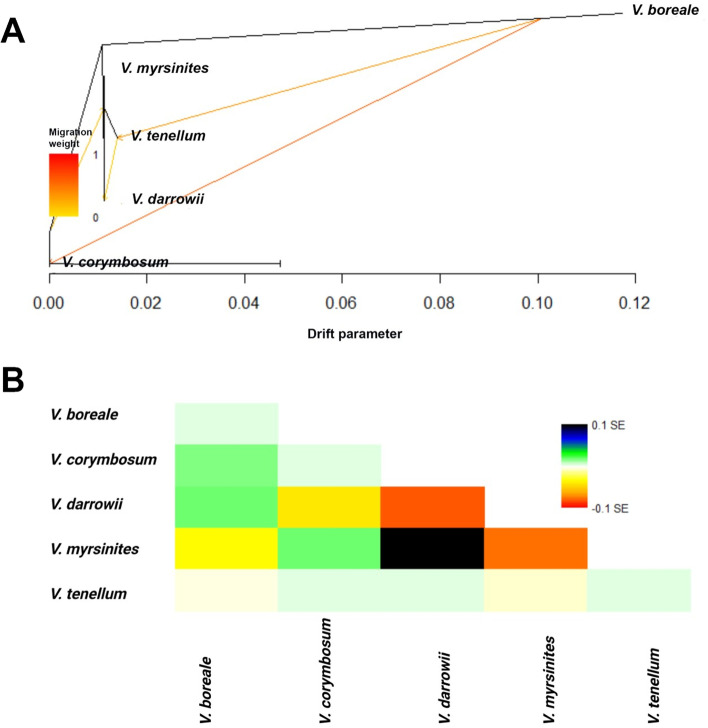
**A** Maximum likelihood (ML) tree of five species of blueberry populations with inferred migration edges. Colored arrows indicate migration events, and the color scale shows the migration weight. **B** Residual fit plotted from the ML tree of five species-wise groups of blueberry populations. A positive residual value indicates that the groups are more closely related

Lineage sorting is an evolutionary trajectory that brings species together and is an essential mechanism for domestication and adaptation. To model lineage sorting between the species, TreeMix analysis was conducted by inferring ancestry via maximum likelihood (ML) analysis that uses genome-wide allele frequencies and then identifies potential gene flow from a residual covariance matrix. The ML tree (Fig. 3A) suggested four migration events between the selected populations. An independent event was shared between *V. boreale* to *V. corymbosum*, thus indicating lateral gene flow with the common ancestor. This event had the highest migration weight of 0.59 (Fig. 3A). Another migration event occurred from *V. boreale* to *V. tenellum*, which had a migration weight of 0.36. These two migration events indicated gene flow from *V. boreale* to the other two species (*V. corymbosum* and *V. tenellum)* and *V. boreale* appears to be ancestral to these two species. The third migration event occurred from *V. tenellum* to *V. darrowii*, with a migration weight of 0.14. The fourth migration event with a migration weight of 0.18 occurred between two subtrees, as shown in the Fig. 3A.

The residual fit of maximum likelihood tree in Fig. 3A is plotted in the Fig. 3B. The results from these plots suggested that *V. darrowii* and *V. myrsinites* are closest to each other than all the other pairs and are hence candidates for admixture events, which we observed in our study. Further, *V. corymbosum* was seen closer to *V. myrsinites* than the *V. darrowii*, and *V. tenellum*.

### Pairwise selective sweep analysis

**Fig. 4:**
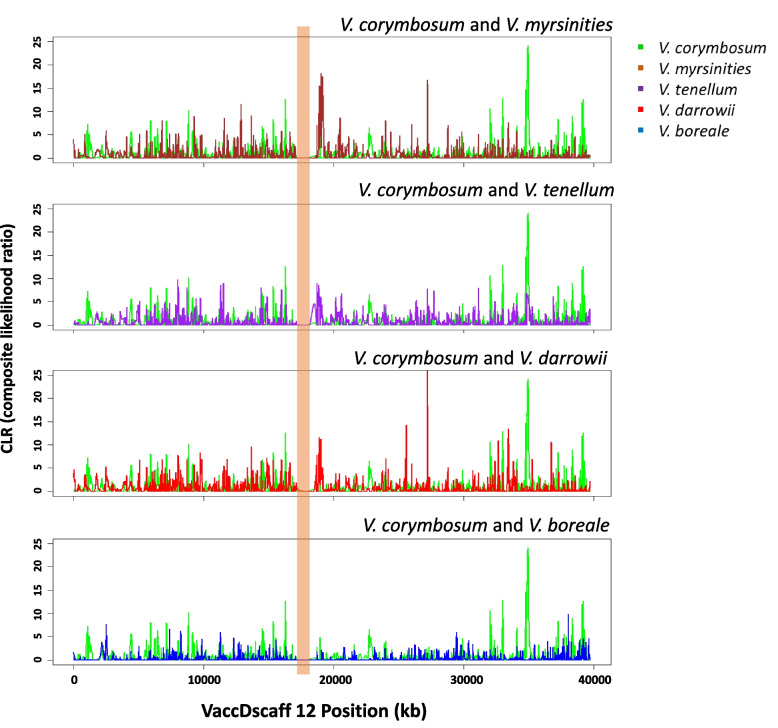
Scan of the scaffold VaccDscaff 12 showing selective sweep. The *x*-axis denotes the position on the scaffold VaccDscaff 12, and the *y*-axis shows the composite likelihood ratio (CLR) evaluated by SweeD. The orange-colored vertical bar shows the selective sweep present in all the four pairwise sweep analyses

To identify genomic loci underlying the domestication of the blueberries, we used pairwise selective sweep analysis and detected signatures of selective sweeps for each species in comparison with *V. corymbosum*. The analysis revealed several genomic regions for all the species (Fig. S4). The most prominent selective sweep detected across all four wild species was the genomic regions on the scaffold VaccDscaff 12 (Fig. 4), spanning 17,197,565 to 17,912,802 bp. This genomic region harbored 32 genes that were annotated in this region. Details of these genes, including *Arabidopsis* homologs and Gene Ontology terms, are given in Table 5. A closer look at the functions of these genes suggests that most of these genes are related to primary metabolic pathways, including biosynthetic and signaling processes. Six of the 32 genes encoded DNA topoisomerase 2, causing topological changes needed to resolve meiotic recombination. Four genes encoded interaptin, and three encoded callose synthase (Table 5). The gene *augustus_masked-VaccDscaff12-processed-gene-172.10*, encoding the protein MAINTENANCE OF MERISTEMS-like (*Arabidopsis* homolog AT2G25010.1), is required to maintain genome stability and cell division activity in meristematic cells [30]. These genes may underlie the molecular genetic basis of domestication or favorable horticultural traits that may have contributed to the improvement in blueberry.

**Table 5:** Summary of the 32 genes, including Gene Ontology terms, from the major selective sweep, detected on the the scaffold VaccDscaff 12

SeqName	Annotation	Length	#Hits	e-Value	*Arabidopsis* homolog
augustus_masked-VaccDscaff12-processed-gene-172.10	Protein MAINTENANCE OF MERISTEMS-like	3737	10	1.83E-34	AT2G25010.1
augustus_masked-VaccDscaff12-processed-gene-173.0	PREDICTED: uncharacterized protein LOC107810502	1137	10	1.28E-11	---NA---
maker-VaccDscaff12-snap-gene-173.17	DNA topoisomerase 2	245	10	4.38E-33	AT3G23890.2
maker-VaccDscaff12-snap-gene-173.19	DNA topoisomerase 2-like	729	10	9.11E-13	AT3G23890.2
maker-VaccDscaff12-snap-gene-173.16	DNA topoisomerase 2	245	10	4.38E-33	AT3G23890.2
maker-VaccDscaff12-snap-gene-173.18	DNA topoisomerase 2-like	239	10	1.12E-15	AT3G23890.2
maker-VaccDscaff12-snap-gene-173.20	---NA---	1007			---NA---
maker-VaccDscaff12-augustus-gene-173.14	---NA---	1900			---NA---
maker-VaccDscaff12-augustus-gene-174.17	DNA topoisomerase 2-like	408	10	3.24E-15	AT3G23890.2
augustus_masked-VaccDscaff12-processed-gene-174.7	Callose synthase 10 isoform X2	18,273	10	1.05E-27	AT2G36850.1
maker-VaccDscaff12-snap-gene-174.22	---NA---	5469			---NA---
maker-VaccDscaff12-snap-gene-174.23	---NA---	5818			---NA---
maker-VaccDscaff12-snap-gene-175.34	Callose synthase 10 isoform X2	8520	10	2.84E-26	AT2G36850.1
maker-VaccDscaff12-snap-gene-175.35	Callose synthase 9	9063	10	6.19E-07	AT2G36850.1
maker-VaccDscaff12-snap-gene-176.26	Uncharacterized protein LOC109834839	7786	10	2.38E-25	AT5G18880.1
augustus_masked-VaccDscaff12-processed-gene-176.2	Uncharacterized protein LOC110900598	3546	10	1.43E-118	AT3G24255.1
augustus_masked-VaccDscaff12-processed-gene-177.2	E3 ubiquitin-protein ligase Praja-2	2090	10	4.96E-24	AT3G02340.1
snap_masked-VaccDscaff12-processed-gene-177.21	Nitrate regulatory gene2 protein isoform X2	7397	10	2.31E-22	AT5G25590.1
augustus_masked-VaccDscaff12-processed-gene-177.4	Uncharacterized protein LOC110779242	2260	10	4.47E-58	AT1G43760.1
maker-VaccDscaff12-snap-gene-178.40	E3 ubiquitin-protein ligase Praja-2-like	379	10	2.21E-28	AT3G02340.1
maker-VaccDscaff12-snap-gene-178.38	---NA---	1070			---NA---
maker-VaccDscaff12-augustus-gene-178.30	Gamma-secretase subunit APH1-like	2021	10	9.72E-14	AT2G31440.1
maker-VaccDscaff12-snap-gene-178.36	Interaptin	2111	10	1.60E-43	AT3G55060.1
augustus_masked-VaccDscaff12-processed-gene-178.1	Interaptin	510	10	1.48E-38	AT3G55060.1
augustus_masked-VaccDscaff12-processed-gene-178.2	Golgin subfamily A member 4	1266	10	3.61E-58	AT3G55060.1
augustus_masked-VaccDscaff12-processed-gene-178.4	DNA topoisomerase 2	363	10	2.63E-37	AT3G23890.1
snap_masked-VaccDscaff12-processed-gene-178.19	Protein MOR1 isoform X1	7003	10	2.52E-07	AT2G42740.1
maker-VaccDscaff12-snap-gene-179.34	Gamma-secretase subunit APH1-like	1952	10	7.83E-14	AT2G31440.1
augustus_masked-VaccDscaff12-processed-gene-179.1	E3 ubiquitin-protein ligase Praja-2-like	9338	10	1.58E-22	AT3G02340.1
maker-VaccDscaff12-snap-gene-179.36	Interaptin	2010	10	4.80E-38	AT3G55060.1
augustus_masked-VaccDscaff12-processed-gene-179.3	Interaptin	510	10	4.67E-40	AT3G55060.1
augustus_masked-VaccDscaff12-processed-gene-179.4	Golgin subfamily A member 4	1335	10	6.75E-58	AT3G55060.1

## Discussion

Introgression with bridge accessions is needed to widen genetic diversity among the blueberries. We performed this study to identify species boundaries and bridge accessions to widen the genetic diversity among the cultivated pool and compare species pairwise to resolve reticulation and gene flow. We used genome-wide SNPs in collections of five critical species historically used for introgression and breeding. The current study determined genetic relatedness and admixture in the selected accessions, which will be helpful to understand the role of introgression and wide hybridization in the US blueberry breeding history. This study allowed us to track lineages in the present-day domesticated *V. corymbosum* cultivars admixed due to interspecific hybridizations.

### Genomic relationships in SHB and NHB accessions

Southern highbush cultivars have been developed by introgressing *V. darrowii* into northern highbush *V. corymbosum* background. The primary goal was to introduce adaptation for lower chill requirements [31]. The phylogenetic analysis based on the nuclear genome-wide SNP data indicated that SHB and NHB are more closely related to each other [21, 32]. In PCA [33]. and phylogenetic analysis (Fig. S3E), we observed that several of the NHB accessions clustered along with SHB cultivars, which could be due to recurrent backcrosses while developing SHB cultivars. Thus, our study resolved the relationship between SHB and NHB [21].

### *Vaccinium boreale* is genetically distant outgroup

*V. boreale* is a small, deciduous shrub growing 1-9 cm tall, spreading by shallow rhizomes to form dense colonies of many individuals. It is native to the northern part of the United States and parts of southeastern Canada. It is mainly restricted to alpine, subalpine (non-forested, upland), or rocky uplands and open conifer forests near high altitudes. The *V. boreale* accessions used in this study were the rhizomes collected from Kennington Cove and Ocean Cobble beach at the edge of Louisbourg National Historical Park in Nova Scotia, Canada. Various analyses in this study place *V. boreale* as an outgroup and share restricted gene flow with the other *Vaccinium* species, primarily *V. corymbosum*. The two strong migration events with migration weights of 0.59, and 0.36 indicated gene flow from *V. boreale* to the *V. corymbosum* and *V. tenellum*, and *V. boreale* appears to be ancestral to these species. This observation was further strengthened by the current admixture analysis revealing that *V. boreale* and *V. corymbosum* share the single clade at K = 2 level but were separated at K = 3 [33]. Interestingly, *V. boreale*, *V. angustifolium,* and *V. myrtilloides* cohabitate across the Allegheny range of Canada indicating this region may be the primary center of diversity.

In this study, admixture analysis revealed that *V. corymbosum* species are genetically related to *V. boreale* and might have shared lineages. Furthermore, the detection of shared ancestry of *V. boreale* in *V. corymbosum* in TreeMix analysis can be considered further evidence for genetic relatedness between these two species. Thus, the migration events could be noted in this study from *V. boreale* to *V. corymbosum* and in the subtrees involving *V. corymbosum* and *V. myrsinites* possibly indicate genetic reticulation involving northern to southern species. More extensive set of collections from each of these species will be needed to place *V. corymbosum* in the phylogenetic network accurately.

### Genetic differentiation within southern blueberry species

An extensive geographic distribution range and outcrossing rates may have significantly contributed to the high genetic diversity observed in southern species- *V. darrowii, V. myrsinites,* and *V. tenellum* [34]. *V. darrowii,* and *V. myrsinites,* were noted in this study as highly diverse, whereas *V. tenellum* accessions had a relatively narrow genetic background. In this study, however, they might be admixed with *V. pallidum/V. vaccillans* in coastal North Carolina, and hence further admixture analysis including these two species will be necessary. The TreeMix analysis indicated gene flow from *V. tenellum* to *V. darrowii,* implying that *V. tenellum* might be ancestral to *V. darrowii*.

Southern blueberry species represent a rich genetic resource for selecting accessions with valuable horticultural traits [34]. Admixed populations resulting from the wide hybridization of *V. darrowii* and *V. myrsinites* are evergreen xerophytic, fire-adapted plants [4, 7, 8] and occupy wide ranges [5]. *V. myrsinites* is probably an autotetraploid of *V. darrowii,* and thus other than chromosome counts or hybridization experiments, grey glaucescence on new growth flushes, and fruit of *V. darrowii* can be used to separate the two species. New growth flushes on *V. myrsinites* are shiny green while immature berries are shiny green or greenish red [35]. Our analysis provides valuable information about genetic reticulation among the selected accessions. Seven *Vaccinium* species, including *V. darrowii, V. corymbosum*, and *V. tenellum*, are known as ancestral to cultivated SHB and NHB genomes [7]. Also, two more species, *V. myrtilloides*, and *V. pallidum*, have been reported to partially contribute to the current gene pool of highbush cultivars [21, 36]. Further research involving wild *V. corymbosum* species will be needed for further understanding of the gene flow among northern and southern species.

### Strong selective sweeps of domestication identified on scaffold 12

Domestication studies is a complex evolutionary process by which cultivars are selected that differ from their wild progenitors in quality, yield, or adaptation [37]. Although the selection is primarily based on a preferred phenotype of interest, such as fruit taste and size, it involves the presence and interactions of genes associated with the desired phenotype selected at the genetic level. Thus, identifying strong signatures can lead to the discovery of candidate genes involved in the domestication processes. However, the positive selection signatures between wild and cultivated blueberry species must be identified to reveal the genes underpinning the evolution of domesticated blueberry *V. corymbosum*. Positive selection accumulates beneficial alleles, shifts allele frequencies in the population, and leaves a signature over time in the genome [38]. Such patterns of advantageous mutations fixed or selected during domestication can be revealed by analyzing extensive genomic data. In this study, a 715-kb region that harbored 32 genes of primary metabolic pathways was a common sweep for selective signatures in all the pairwise comparisons. This selected region might relate to domestication, including adaptation to a particular climate and favorable fruit traits. Many candidate genes from this region were involved in primary metabolism. Thus, further functional genomic analysis of these genes would have great significance in understanding the domestication process of cultivated blueberries.

## Conclusion

The present study demonstrated that GBS is reliable for identifying high-quality SNPs for investigating the genetic relatedness of blueberry species. The identified genome-wide SNPs in the selected blueberry accessions were successfully mapped to the tetraploid Draper reference genome and used to elucidate the genetic structure. Also, we resolved that *V. boreale* is genetically distinct from the other species. We further identified migration events that provided insights into the evolutionary trajectories important for domestication and adaptation. The genomic region of the selective sweep identified on the scaffold VaccDscaff 12 comprised 32 genes, which could be crucial for the domestication of cultivated blueberries. Furthermore, PCA, phylogenetic, and admixture analyses resolved shared genetic lineages revealing collections of *V. myrsinites and V. darrowii* are highly admixed and do not exhibit distinct species boundaries. The observations made in this study may help understand the genetic relationships among the related species, enhance the breeding of horticultural traits in cultivated blueberries.

## Materials and methods

### Plant materials

A total of 195 blueberry accessions (Tables S2, S3, Fig. S5) were used in this study. These individuals represented the following species: *V. tenellum* (*N* = 38), *V. darrowii* (*N* = 81), *V. myrsinites* (*N* = 29), *V. boreale* (*N* = 14), and *V. corymbosum* (*N* = 33). *V. myrsinites* and *V. corymbosum* (except NJOPB-8) accessions are tetraploid (2n = 4x = 48), and *V. tenellum, V. darrowii*, and *V. boreale* accessions are diploid (2n = 2x = 24). The NJOPB-8 is a diploid *V. corymbosum* clone originated from Burlington County, New Jersey. The plants were collected across North America in 1980s and 1990s, propagated, and maintained under greenhouse conditions at Philip E. Marucci Center for Blueberry & Cranberry Research and Extension managed by Rutgers, the State University of New Jersey. For DNA extractions, only a few young leaves were collected in 15-mL centrifuge tubes, kept in dry ice, transferred to Delaware State University, and stored at − 80 °C for later use. We confirm that IUCN Policy Statement includes all methods as per the Convention on the Trade in Endangered Species of Wild Fauna and Flora. Since these are perennial plants, all the above accessions remain at Philip E. Marucci Center for Blueberry & Cranberry Research and Extension.

### DNA extraction

The homogenization of the leaf tissue and DNA extraction were performed as described in [33]. Leaf tissue ranging from 100 to 120 mg was placed in a 2-ml tube containing a metallic bead. The tissue samples were crushed by using TissueLyzer-II (Qiagen, USA) and used in DNA extraction, performed using the DNeasy mini-plant and DNA plant pro kits (Qiagen, USA) following the manufacturer’s instructions. The DNA purity was verified with a Nanodrop spectrophotometer (Thermo Fisher Scientific, Waltham, MA, USA) and quantified with both Nanodrop and Qubit (Thermo Fisher Scientific, Waltham, MA, USA). The quality of each DNA sample was also verified in 1% agarose gels, stained with ethidium bromide, and visualized under UV light. Samples were stored at − 20 °C.

### Library preparation, sequencing, and data analysis

DNA samples were normalized to a final concentration of 10 ng/μl and GBS was performed with the *ApeK*I restriction enzyme [39]. The library preparation and post-sequencing analysis were performed according to established protocols. The library’s quality and quantification were validated using the Bio-analyzer 2100 (Thermo Fisher Scientific, Waltham, MA, USA) and Qubit 4 fluorimeter (Thermo Fisher Scientific, Waltham, MA, USA). The library was sequenced by using the NextSeq500 platform with paired-end sequencing chemistry. Sequencing reads were aligned to the *Vaccinium corymbosum* cv. 1 Draper (tetraploid) v1.0 genome sequence ( [28]; http://gigadb.org/dataset/100537). The first 12 scaffolds were used as a reference to align the reads. SNP calling was performed using GB-eaSy (https://github.com/dpwickland/GB-easy). The SNPs obtained were filtered with minor allele frequency (MAF) > 0.05%, missing data (call) rate > 90%, and read depth (DP) > 3 as criteria before analysis.

### Genetic divergence and population structure analyses

Genetic diversity values were calculated by a neighbor-joining algorithm using TASSEL 5 (www.maizegenetics.net). The EIGENSTRAT algorithm [40] with the SNP and Variation Suite (SVS v8.8.5; Golden Helix, Bozeman, MT, USA, www.goldenhelix.com) was used for Principal Component Analysis (PCA). Observed nucleotide diversity (π) for various chromosomes was estimated with sliding-window analysis by using TASSEL v5.0 [41]. The fixation index (*F*_*ST*_) was calculated by using Wright’s F statistic [42] with SVS v8.8.5 (Golden Helix, Bozeman, MT, USA, www.goldenhelix.com).

### Admixture analysis

The admixture analysis involved using a least-squares optimization approach implemented in the sNMF function of the R package LEA [29, 43]. The number of clusters obtained resulted from the cross-entropy criterion, based on the prediction of a fraction of masked genotypes (matrix completion), and the cross-validation approach. The number of K populations was evaluated from 1 to 6 clusters, with ten replications performed for each run. The best K value was chosen when the cross-entropy curves exhibited a plateau.

### Detection of pairwise selective sweeps

We used an open-source tool, SweeD 4.0.0 [44], to analyze the site frequency spectrum (the distribution of the expected number of polymorphic sites), for pairwise selective sweep analysis. The selective sweeps between *V. corymbosum* and each of the wild blueberry species were identified based on the composite likelihood ratio tests. The genes in the selective sweep regions were retrieved from the Draper genome sequence gff file (http://gigadb.org/dataset/100537) using the coding sequence coordinates [28]. The obtained FASTA sequences were annotated using NCBI and TAIR databases to obtain the protein information and Gene Ontology annotation terms (biological process, molecular function, and cellular component). The *Arabidopsis* homologs were added to understand the annotations of the genes from the selective sweep regions.

### TreeMix analysis

To model the gene flow and identify the migration events between the selected blueberry species, we used TreeMix22 v.1.12 [45]). The programs infer population splitting and mixing patterns from genome-wide allele frequency data. For a given set of allele frequencies, the program will return the maximum likelihood tree for the collection of populations and attempt to infer potential gene flow from the residual covariance matrix. In this study, we used five migration events for modeling.

## Supplementary Information

**Additional file 1: Table S1.** SNPs showing the highest FST indices across the scaffolds.

**Additional file 2: Table S2.** List of blueberry wild accessions used in this study.

**Additional file 3: Table S3.** List of *Vaccinium corymbosum* cultivars used in this study (Kulkarni et al. 2020. Int. J. Mol. Sci., 22(1):163; https://doi.org/10.3390/ijms22010163).

**Additional file 4: Fig. S1.** PCA plots depicting the distribution of wild blueberry accessions against *V. corymbosum*.

**Additional file 5: Fig. S2.** Manhattan plots of the genome-wide window-based overall and pairwise *F*_*ST*_ (fixation index) values for 195 accessions across the 12 scaffold. A. Pairwise *F*_*ST*_ analyses showing positive selection. B. Pairwise *F*_*ST*_ analyses showing negative selection. The significant thresholds (red line) are set at the top (for positive *F*_*ST*_ scores suggesting signature of positive selection) or bottom (for negative *F*_*ST*_ scores suggesting signature of negative selection) at 0.2% of the *F*_*ST*_ distribution.

**Additional file 6: Fig. S3.** Cluster analysis based on the genetic distance using a Neighbor-Joining (NJ) tree revealing intraspecific genetic variation within *V. tenellum* (A) *V. boreale* (B), *V. myrsinites* (C), *V. darrowii* (D), and *V. corymbosum* (E).

**Additional file 7: Fig. S4.** Signatures of selective sweeps for each species in comparison with *V. corymbosum* detected on all the scaffolds in pairwise selective sweep analysis.

**Additional file 8: Table S4.** Eigenvalues for the first two principal components estimated for selected blueberry accessions.

**Additional file 9: Fig. S5.** Geographical map of the US and Canada showing the sites from which the accession were collected.

## Data Availability

The raw reads generated from the genotyping-by-sequencing in the present study are available at NCBI under the bioProject accession number: PRJNA900612 (https://www.ncbi.nlm.nih.gov/bioproject/PRJNA900612/).

## Abbreviations

*F*_*ST*_: Fixation index
GBS: Genotyping-by-sequencing
ML: Maximum likelihood
MAF: Minor allele frequency
NJ: Neighbour-joining
NHB: Northern highbush blueberry
PCA: Principal component analysis
DP: Read depth
SNP: Single nucleotide polymorphism
SHB: Southern highbush blueberry
