# Supplementary figures and images for: Genomic and evolutionary relationships among wild and cultivated blueberry species
Source: BMC Plant Biol. 2023 Mar 6;23:126. doi: 10.1186/s12870-023-04124-y (PMC9987114; doi:10.1186/s12870-023-04124-y)

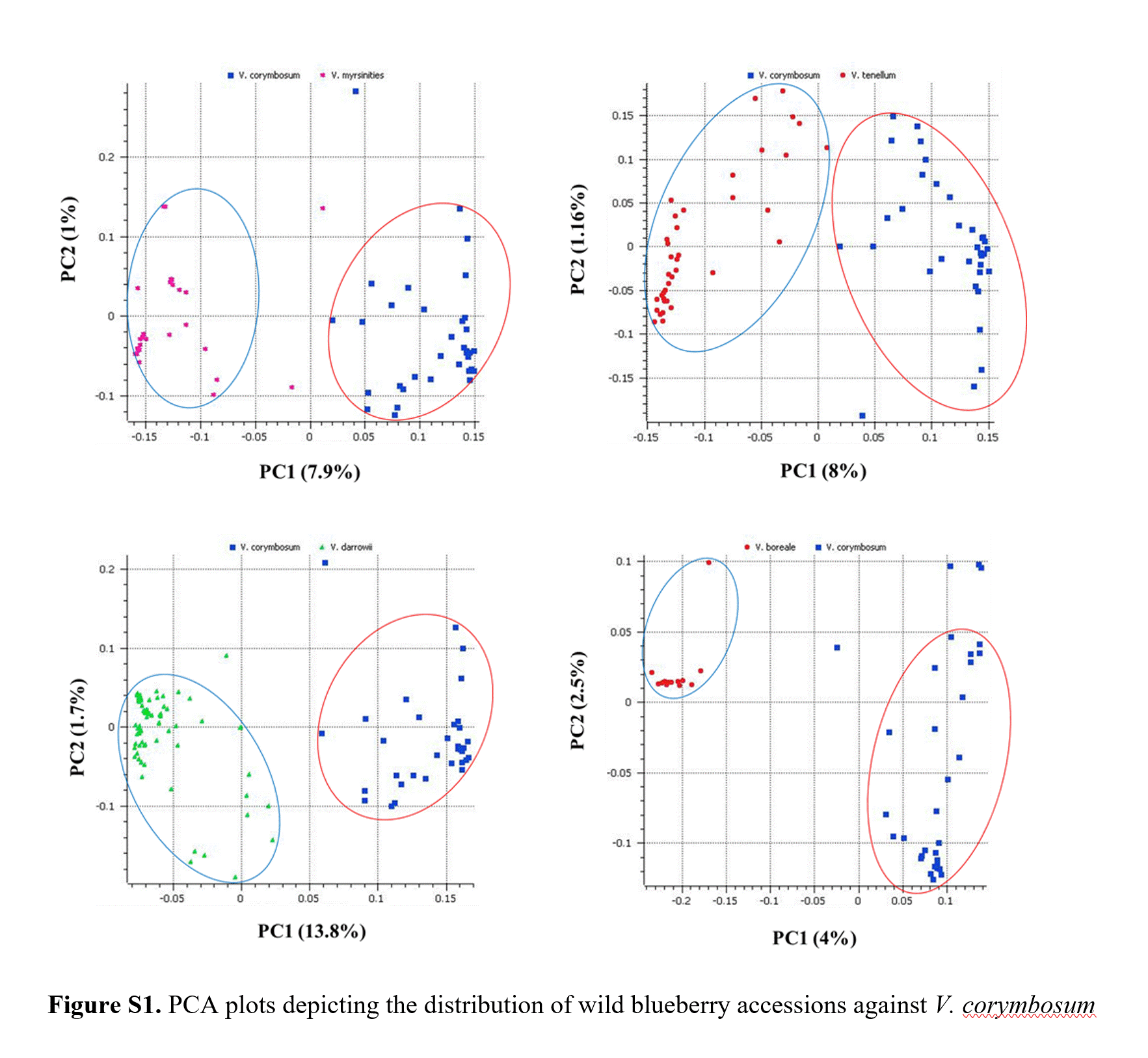

Supplement: Supplementary file 4 — Additional file 4: Fig. S1. PCA plots depicting the distribution of wild blueberry accessions against V. corymbosum. [file 12870_2023_4124_MOESM4_ESM.gif]

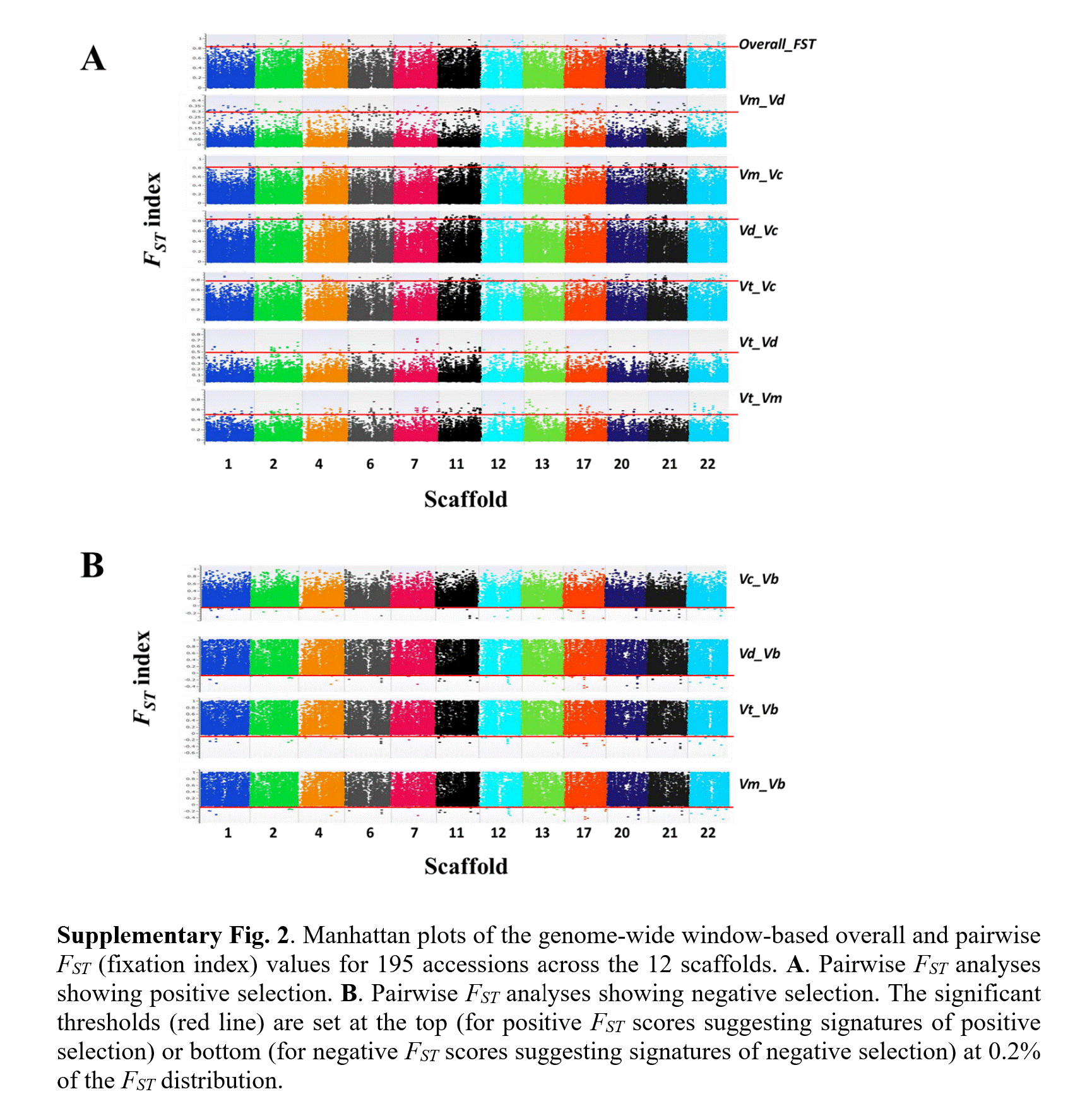

Supplement: Supplementary file 5 — Additional file 5: Fig. S2. Manhattan plots of the genome-wide window-based overall and pairwise FST (fixation index) values for 195 accessions across the 12 scaffold. A. Pairwise FST analyses showing positive selection. B. Pairwise FST analyses showing negative selection. The significant thresholds (red line) are set at the top (for positive FST scores suggesting signature of positive selection) or bottom (for negative FST scores suggesting signature of negative selection) at 0.2% of the FST distribution. [file 12870_2023_4124_MOESM5_ESM.gif]

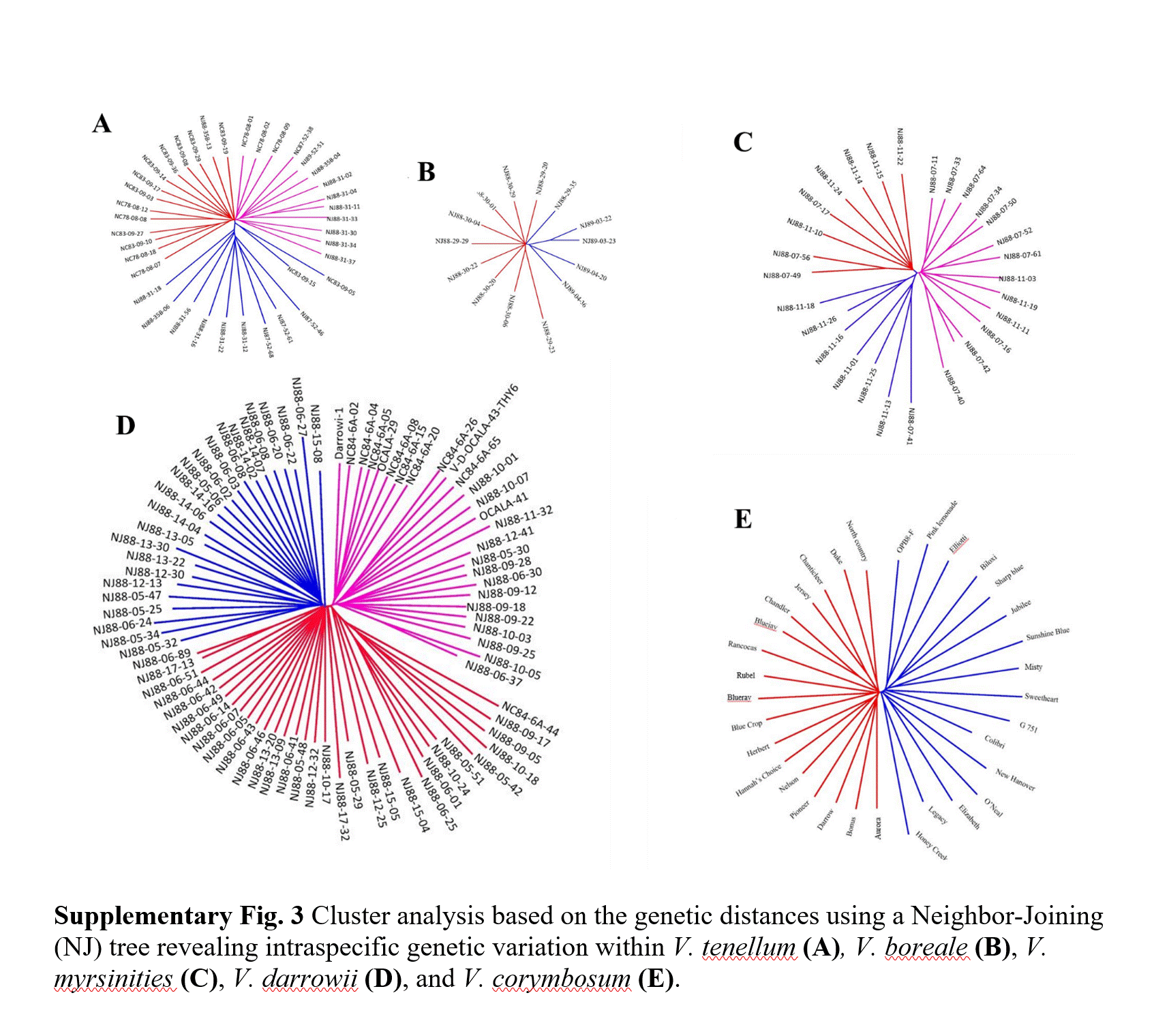

Supplement: Supplementary file 6 — Additional file 6: Fig. S3. Cluster analysis based on the genetic distance using a Neighbor-Joining (NJ) tree revealing intraspecific genetic variation within V. tenellum (A) V. boreale (B), V. myrsinites (C), V. darrowii (D), and V. corymbosum (E). [file 12870_2023_4124_MOESM6_ESM.gif]

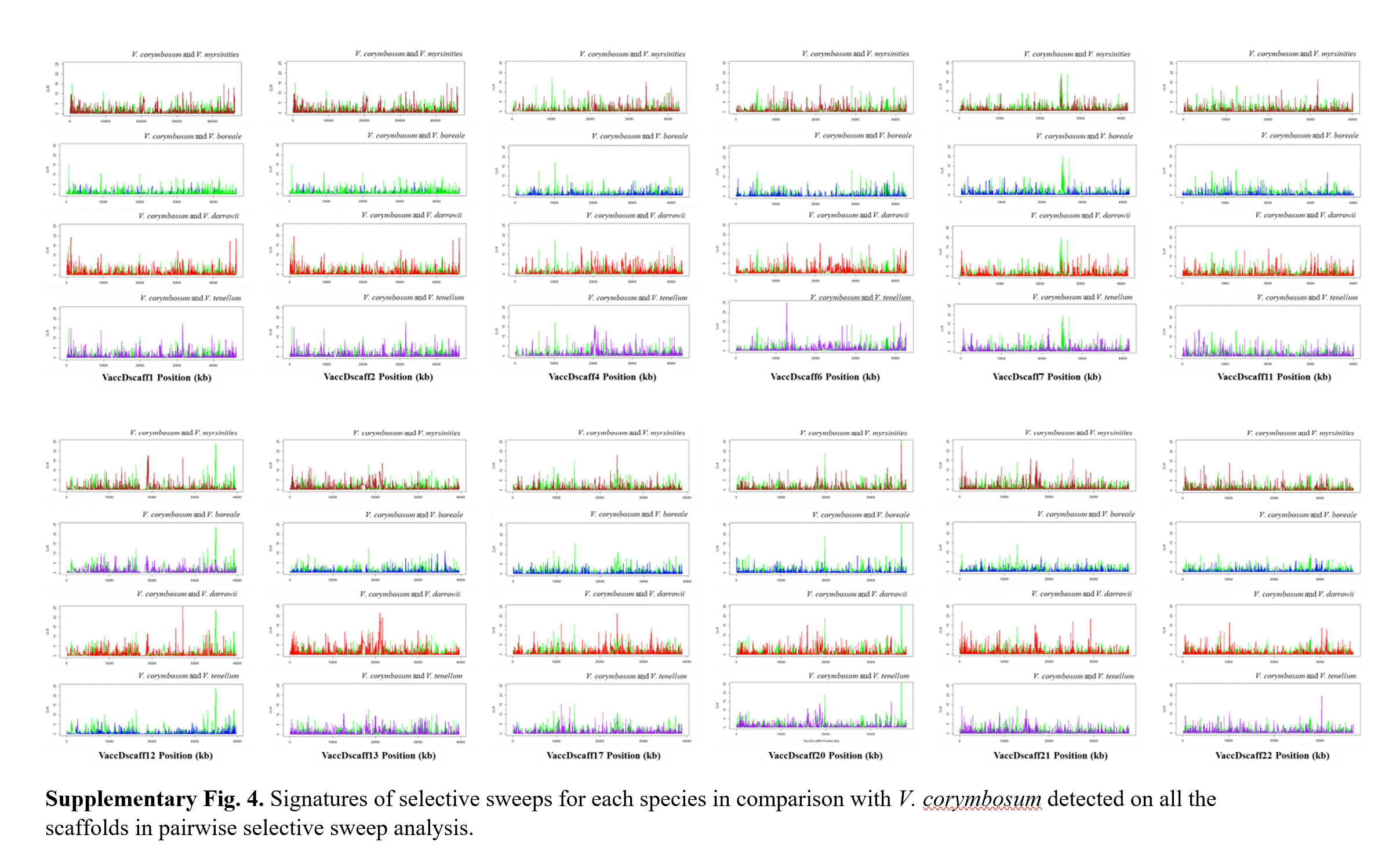

Supplement: Supplementary file 7 — Additional file 7: Fig. S4. Signatures of selective sweeps for each species in comparison with V. corymbosum detected on all the scaffolds in pairwise selective sweep analysis. [file 12870_2023_4124_MOESM7_ESM.gif]

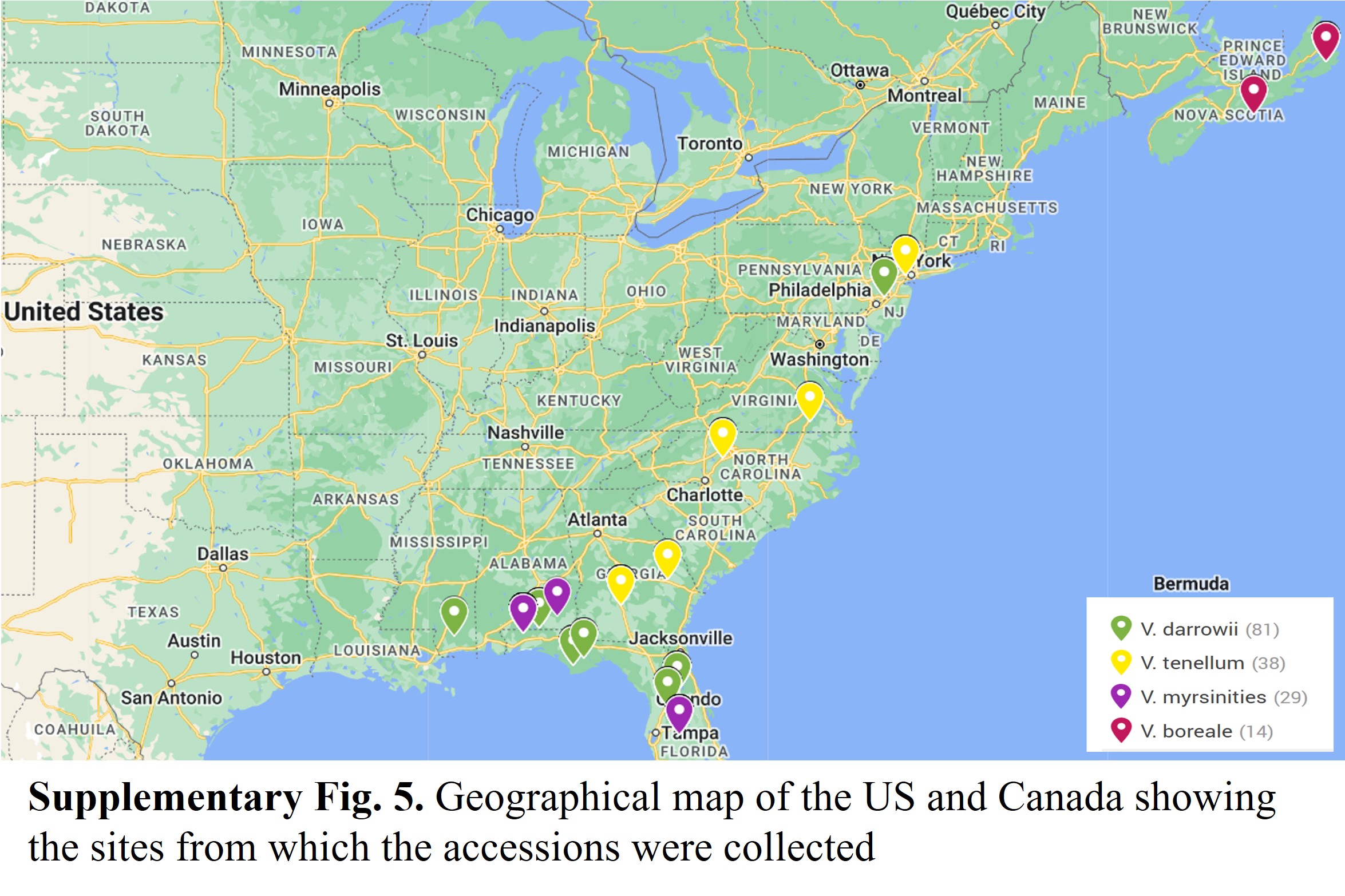

Supplement: Supplementary file 9 — Additional file 9: Fig. S5. Geographical map of the US and Canada showing the sites from which the accession were collected. [file 12870_2023_4124_MOESM9_ESM.jpg]
